# Resting Cyst Distribution and Molecular Identification of the Harmful Dinoflagellate *Margalefidinium polykrikoides* (Gymnodiniales, Dinophyceae) in Lampung Bay, Sumatra, Indonesia

**DOI:** 10.3389/fmicb.2019.00306

**Published:** 2019-02-21

**Authors:** Hikmah Thoha, Muawanah, Mariana D. Bayu Intan, Arief Rachman, Oksto Ridho Sianturi, Tumpak Sidabutar, Mitsunori Iwataki, Kazuya Takahashi, Jean-Christophe Avarre, Estelle Masseret

**Affiliations:** ^1^Research Center for Oceanography, Indonesian Institute of Sciences, Jakarta, Indonesia; ^2^Main Center for Marine Aquaculture of Lampung, Directorate General of Aquaculture, Lampung, Indonesia; ^3^Asian Natural Environmental Science Center, The University of Tokyo, Tokyo, Japan; ^4^ISEM, IRD, CNRS, EPHE, University of Montpellier, Montpellier, France; ^5^MARBEC, University of Montpellier, IRD, Ifremer, CNRS, Montpellier, France

**Keywords:** *Margalefidinium polykrikoides*, dinoflagellate, harmful algal blooms, resting and hyaline cysts, Indonesia

## Abstract

*Margalefidinium polykrikoides*, an unarmored dinoflagellate, was suspected to be the causative agent of the harmful algal blooms – associated with massive fish mortalities – that have occurred continually in Lampung Bay, Indonesia, since the first bloom event in October 2012. In this study, after examination of the morphology of putative *M. polykrikoides*-like cysts sampled in bottom sediments, cyst bed distribution of this harmful species was explored in the inner bay. Sediment samples showed that resting cysts, including several morphotypes previously reported as *M. polykrikoides*, were most abundant on the northern coast of Lampung Bay, ranging from 20.6 to 645.6 cysts g^-1^ dry sediment. Molecular phylogeny inferred from LSU rDNA revealed that the so-called Mediterranean ribotype was detected in the sediment while *M. polykrikoides* motile cells, four-cell chain forming in bloom conditions, belonged to the American-Malaysian ribotype. Moreover, hyaline cysts, exclusively in the form of four-cell chains, were also recorded. Overall, these results unequivocally show that the species *M. polykrikoides* is abundantly present, in the form of vegetative cells, hyaline and resting cysts in an Indonesian area.

## Introduction

The distribution and frequency of harmful algal blooms (HABs), as well as their negative effects such as shellfish poisoning syndromes, have dramatically increased in recent decades (Hallegraeff, 1993; Maso and Garcés, 2006; Manfrin et al., 2012). Among the HAB causative agents, the harmful unarmored dinoflagellate *Margalefidinium polykrikoides* (Margalef, 1961) Gómez et al. (2017) (Gymnodiniales) shares many features with an invasive species: cosmopolitanism, geographic discontinuity and a wide ecological spectrum. This species, initially named *Cochlodinium polykrikoides* Margalef, groups in different phylogenetic clades according to its ribotype, and these clades usually correlate with the supposed geographic origin of the strains (Iwataki et al., 2008). Accordingly, *C. polykrikoides* was divided into four groups and two clades based on the phylogeny of D1–D2 domain of LSU rDNA (Reñé et al., 2013). Then, following extensive morphological and molecular studies, *C. polykrikoides* was recently renamed *M. polykrikoides* Margalef (Gómez et al., 2017).

*Margalefidinium polykrikoides* has a complex life cycle that is not entirely known, with a pelagic and a benthic phase. It produces resting cysts after sexual reproduction (Tang and Gobler, 2009; Li et al., 2015), which correspond to the benthic stage. It is also able to form immotile chain-forming cells surrounded by a transparent membrane, called hyaline cysts (Kim et al., 2002). Resting cysts are deposited in the sediment (Li et al., 2015 and references therein). The most plausible source of dispersal of cyst-producing dinoflagellates is via ballast waters or shellfish transfers (Bolch and de Salas, 2007; Laabir et al., 2007). Kudela and Gobler (2012) compiled a complete inventory of the expansion and ecological strategies of *M. polykrikoides*. Before 1990, its blooms had mainly been observed in Japan and in Central and North America (Matsuoka et al., 2008; Iwataki et al., 2015). As of the early 1990s, it spread to South Korea, where the fisheries sector experienced annual losses of over USD 100 million (Kim, 1997). Since then, its blooms have been visible throughout Asia, in Europe, and in North America. As of late 1990s, this species has also been observed on the coasts of Italy (Sannio et al., 1997; Siano et al., 2002; Zingone et al., 2006) as well as in the Black Sea, in Odessa (Terenko, 2005) and in Cape Utrish (Vershinin et al., 2004; Vershinin et al., 2005), where it causes extensive blooms. Although *M. polykrikoides* has rarely been observed on the Catalonian (northeastern Spanish Mediterranean) coast, concentrations of up to 2 × 10^4^ cells L^-1^ were recorded in 2011 in the port of Arenys, North of Barcelona (Reñé et al., 2013). Until recently, three sub-clades, or ribotypes, of *M. polykrikoides* had been identified: the East-Asian ribotype (which refers to the original Japanese-Korean clade), the American-Malaysian ribotype, and the Philippines ribotype (Iwataki et al., 2008). However, comparison of the partial large subunit of the ribosome (LSU rDNA) gene sequence from several individuals sampled on the Catalan coast in 2011 and 2012 suggested that most populations of *M. polykrikoides* formed a new “Mediterranean” ribotype, the others belonging to the Philippines ribotype (Reñé et al., 2013).

In Asia, where intensive marine aquaculture strives to satisfy the increasing need for food, thousands of seafood poisonings have been attributed to HABs (Corrales and Maclean, 1995; Nicolas et al., 2017). Indonesia, with more than 250 million inhabitants, has a vast maritime territory, with nearly 108,000 km of coastline. Although no detailed information is available, the number of HAB events has been growing in recent years and now affects many Indonesian coastal waters, especially in semi-confined bays that experience high anthropogenic pressures such as Jakarta Bay, Ambon Bay, Kao Bay, and Lampung Bay (Wiadnyana et al., 1996; Matsuoka et al., 1999; Sidharta, 2004, 2005; Aditya et al., 2013). Lampung Bay, located southeast of the island of Sumatra, facing the Sunda Strait, hosts numerous economic activities such as finfish and shellfish aquaculture, pearl farming, fishing and port activities (Sachoemar et al., 2006; Damar et al., 2012). In October 2012, the surface coastal waters off Bandar Lampung, the major city of the bay, became dark brown. The phytoplankton bloom progressively spread to the southern part of the bay. In November 2012, massive fish deaths occurred in many fish farms in Lampung Bay, resulting in economic losses of IDR 850,000,000 (≈USD 60,000), a considerable sum for Indonesian small-scale fish farmers (Muawanah et al., 2013). After microscopic observations, it appeared that the unarmored dinoflagellate *Margalefidinium* was abundant in the water column [Main Center for Marine Aquaculture of Lampung (BBPBL) monitoring observations]. Based on the position of the sulcus and shape of chloroplasts, the possible causative species was identified as *M. polykrikoides* and was clearly differentiated from the similar species *Cochlodinium fulvescens* Iwataki, Kawami and Matsuoka, which had previously been reported in Hurun Bay, a semi-enclosed bay part of Lampung Bay (Iwataki et al., 2007, 2010, 2015). Observed cell concentrations reached up to 110 × 10^6^ cells L^-1^ (BBPBL monitoring unpublished data). Brownish patches appeared gradually in shallow areas and became streaks/plumes that invaded the entire inner bay even to small, remote coves (Emiyati et al., 2017). Since this bloom, phytoplankton communities in Lampung Bay were surveyed weekly by the Main Center for Marine Aquaculture of Lampung (Balai Besar Perikanan Budidaya Laut, BBPBL). This monitoring indicated that the toxic events associated with *M. polykrikoides* have been frequent (BBPBL communication). One hypothesis to explain the sudden appearance and subsequent recurrence of bloom events may be the presence of numerous *M. polykrikoides* cyst beds in bottom sediments. To test this hypothesis, we investigated the presence of cysts of this harmful species in the bottom sediment collected in the inner part of the Lampung Bay. Morphological and molecular identifications of planktonic and benthic cells were performed from the water column and from sediments.

## Materials and Methods

### Studied Area

**FIGURE 1:**
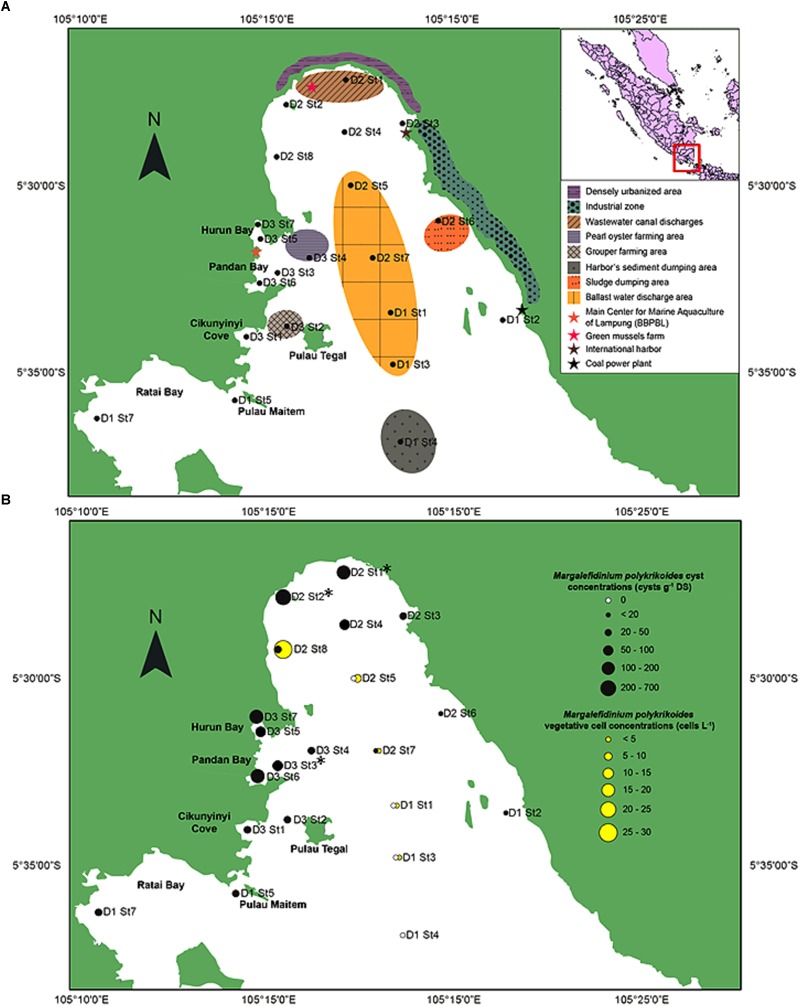
Study area of the southeastern coast of Sumatra, Indonesia. **(A)** Location of the 21 sampling sites with the shore characteristics and human activities of the inner part of Lampung Bay. **(B)** Spatial distribution and densities of *Margalefidinium polykrikoides*-like cysts in the surface sediment (black circles) and of *Margalefidinium polykrikoides* vegetative cells (yellow circles) sampled in May 2014. ^∗^Indicate the stations from which sequences were obtained.

Lampung Bay is located in the southeast of the island of Sumatra, facing the Sunda Strait (Indian Ocean, Indonesia). Bandar Lampung (05.4292 S; 105.2611 E) is the capital and a major economic hub of the Lampung province with a population of 1,451,737 inhabitants in 2017. For this study, twenty-one sampling sites (Figure 1A and Supplementary Table S1) were chosen according to (i) previous observations of HAB events conducted between October 2012 and May 2014 by the Main Center for Marine Aquaculture of Lampung (BBPBL), (ii) the natural features of Lampung Bay (topography, water depth, and sediment characteristics) (Helfinalis, 2000) and (iii) the anthropogenic activities which occur in and/or affect the bay: finfish and shellfish farming, shipping, port activities and wastewater discharges from the watershed (Damar, 2003; Sachoemar et al., 2006).

### Sediment and Phytoplankton Sampling

Three sampling campaigns were realized between 2014 and 2016. The major one was conducted on May 13–15, 2014. Surface sediment samples were collected using an Ekman grab sampler. The upper layer of sediment samples (0–10 cm) was carefully laid in plastic boxes and stored in the dark at 6°C until further analysis. Phytoplankton samples were collected using a plankton net (mesh size: 20 μm) at each sampling site and on the same date as sediment samples. Seawater samples were also collected using a modified 1 L Nansen bottle. Phytoplankton cells were preserved with Lugol’s iodine (Elder and Elbrächter, 2010). Temperature, pH, salinity and dissolved oxygen were also measured, as well as sediment water contents (Supplementary Table S1). In order to compare cyst densities with other published data, they were expressed as cysts g^-1^ of dry sediment (DS). Sediment samples (≈2 g) were dried at 105°C for 24 h. Before measurement, each beaker was predried at 450°C for 24 h. The water content was calculated as follow: % Water = [(Ww-Wd)/Ww] × 100, where Ww stands for wet weight and Wd for dry weight.

Two other samplings were realized during two later blooms. In October 2014, seawater was collected at D3 St1 and D2 St2, two regularly affected stations, for *M. polykrikoides* motile cell isolation and cultivation. Finally, a last sampling occurred in August 2016, during which seawater was collected in at D3 St5 in Hurun Bay for *M. polykrikoides* motile cell and hyaline cyst observation.

### Cyst Extraction and Identification

Resting cysts were extracted from sediment by centrifugation on a density gradient of Ludox CL-X colloidal silica (Sigma-Aldrich) modified from Erard-Le Denn and Boulay (1995) and Genovesi et al. (2007). Aliquots (1 g of wet sediment) were suspended in a 24% sucrose (w/v) solution and sonicated for 3 min in a bath sonicator (Bransonic). Suspensions were sieved through 125 and 20 μm meshes. The slurry remaining on the 20 μm mesh was washed with a 24% sucrose (w/v) solution, re-suspended in 20 mL of the same sucrose solution and then covered with 20 mL of Ludox CL-X colloidal silica (Sigma-Aldrich). The tubes were centrifuged for 15 min at 1500 *g*. The supernatant layer containing the resting cysts was removed with a Pasteur pipette, sieved on a 20 μm nylon membrane and washed with 0.2 μm filtered and autoclaved seawater in the dark at 4°C. Numeration and identification were performed under a phase-contrast inverted microscope (Nikon Diaphot 300) with a Sedgewick-Rafter counting cell (PYSER-SGI). Four replicates were prepared for each sediment sample and pooled. Taxonomic identification of the cysts was carried out using identification keys of Matsuoka and Fukuyo (2000), the MARUM website^1^ (Zonneveld and Pospelova, 2015) and photos from the following references (Kim et al., 2007; Tang and Gobler, 2012; Li et al., 2015; Thoha et al., 2015). Cyst abundances were expressed in cysts g^-1^ of DS.

### *M. polykrikoides* Planktonic Cell Identification and Numeration

Identification and numeration of *M. polykrikoides* cells from samples collected in May 2014 were done using a Nikon high-power dissecting stereo-microscope with a Sedgwick-Rafter counting cell. Numeration was performed on a fraction of each water sample (usually 1 mL, i.e., 1:100 of the total volume) and was based on the number of cells (not the number of chains). Phytoplankton identification was carried out according to the following references: (Cupp, 1943; Davis, 1955; Shirota, 1966; Yamaji, 1966; Tomas, 1997; Praseno and Sugestiningsih, 2000; Taylor et al., 2007; Nontji, 2008; Al-Kandari et al., 2009; Al-Yamani and Saburova, 2010; Omura et al., 2012). The number of vegetative cells was then calculated as follows: N = n× 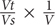, where N is the total number of cells, Vt the volume of the sample, Vs the volume of the subsample, V the volume of the filtered seawater and n the number of observed cells.

*Margalefidinium polykrikoides* cells sampled in October 2014 and hyaline cysts sampled in August 2016 were observed under a Zeiss Axioskop 2 light microscope (Carl Zeiss, Göttingen, Germany) fitted with a Zeiss Axiocam HRc digital camera (Carl Zeiss, Göttingen, Germany). Eight monoclonal cultures were established in 1/2 IFK medium (Wako, Japan) from vegetative cells (LM5D342, 343, 344, 345, 346, 347 from D3 St1 and LM2D348 and LM2D349 from D2 St2) sampled in October 2014.

### Molecular Identification of *M. polykrikoides* From Sediment and Water Samples

Total cyst extracts (10–16 mL) obtained as described above were centrifuged at 12,000 rpm for 10 min at room temperature, and supernatants were discarded. Then, genomic DNA extraction and purification were performed with the PowerLyzer^®^ PowerSoil^®^ DNA Isolation Kit (MO Bio Laboratories, Inc.), according to the manufacturer’s protocol. Briefly, pellets were resuspended in 750 μL of bead solution and transferred to PowerLyzer^®^ glass bead tubes. After gentle vortexing, 60 μL of the lysis solution C1 was added. Samples were vortexed briefly, placed in the PowerLyzer 24 homogenizer^®^ and vortexed again at 4000 rpm for 10 min. The next steps were carried out according to the manufacturer’s protocol. The content of the glass bead tubes was examined under an inverted microscope to check if cyst walls had ruptured correctly. Purified genomic DNA was quantified using a spectrophotometer (NanoDrop 1100).

To assess the presence of *M. polykrikoides* in cyst extracts, several nested primers were designed within the D1–D2 region (Scholin et al., 1994) using Primer3 (Koressaar and Remm, 2007; Untergasser et al., 2012) and synthesized by Eurofins Genomics; their sequences are given in Table 1. Amplifications were carried out in a GeneAmp 2400 PCR cycler (Applied Biosystems), using the KAPA 2G Fast Ready-Mix kit (Kapa Biosystems). For the first round of amplification, each reaction contained 5 μL of 2× Mastermix, 0.4 μM of D1R and D2C primers (Scholin et al., 1994), 1 μL of BSA (0.1 μg/μL final concentration) and 1 μL of genomic DNA, in a final volume of 10 μL. Cycling conditions consisted of an initial denaturation at 95°C for 5 min, followed by 45 cycles of amplification at 95°C for 15 s, annealing at 55°C for 15 s, and elongation at 72°C for 5 s. Amplification products were diluted 1:10 with ultrapure H_2_O and underwent a second round of PCR using a pair of nested primers. Reactions were prepared as described above, using 0.5 μL of diluted amplified DNA, and cycling conditions were the same except that the annealing temperature was 65°C (Table 1). Amplification success was verified by gel electrophoresis with 1.5% (w/v) agarose (Dutscher Scientific) in Tris-acetate EDTA and visualized with SYBR Safe DNA gel stain (Invitrogen) under a blue light transilluminator in the presence of molecular weight markers (Trackit 1 kb plus DNA ladder, Invitrogen). Amplicons were excised from the electrophoresis gel and purified using the QIAquick gel extraction kit (Qiagen). The eluted DNA fragments were sequenced by Macrogen (South Korea). Positive controls consisted of synthetic fragments (Eurofins Genomics) of the partial D1–D2 region from the following *M. polykrikoides* sequences (GenBank accession numbers): AB295048 (American-Malaysian ribotype), AB295046 (Philippines ribotype), AB288383 (East-Asian ribotype), and KC577591 (Mediterranean ribotype); a synthetic fragment of *C. fulvescens* (AB288382) was used as negative control.

**Table 1:** Primers used for *M. polykrikoides* DNA amplifications on motile cells and cyst extracts.

Primer names	Sequences 5′–3′	Annealing temperature	Source
D1R	ACCCGCTGAATTTA AGCATAF	55°C	Scholin et al., 1994
D2C	CCTTGGTCCGTGTT TCAAGAR	55°C	Scholin et al., 1994
CpolyG-F	ACGAACAGGGAAGA GCTCAGF	65°C	This study
Cpoly-R1	GATTGGTTCTCG CGTTGCR	65°C	This study
Cpoly-R2	CCACACGGAGAAA GCAAGTTR	65°C	This study
Cpoly-R3	GAAGTCGTTCGCCG GTTACR	65°C	This study
9R (28-1483R)	GCTACTACCACCAAG ATCTGCR	55°C	Daugbjerg et al., 2000

To determine the LSU sequences from four to five colonies of the eight monoclonal cultures obtained in October 2014, cells of strains LM5D342, 343, 344, 345, 346, 347, LM2D348 and LM2D349 were placed in distilled water to disrupt cells. Cell lysates were directly used for PCR amplification of the D1–D3 region using D1R (Scholin et al., 1994) and 9R (28-1483R) (Daugbjerg et al., 2000) primers and the Ex Tap polymerase (Takara, Japan), following the conditions described by Daugbjerg et al. (2000) and Takahashi et al. (2015). The resulting PCR products were directly sequenced by Eurofins Genomics (Japan).

### Phylogenetic Analyses

Phylogenetic positions of sequences obtained from Lampung Bay were inferred from neighbor-joining (NJ) and maximum likelihood (ML) analyses calculated in Mega 6 (Tamura et al., 2013). Sequences obtained from motile cells and from cyst extracts were aligned with those of *M. polykrikoides* (previously *C. polykrikoides*) available in GenBank. The best base substitution model selected in MEGA for ML analysis was the Tamura-Nei model plus gamma shape parameter (= 1.0971). Two *C. fulvescens* sequences (AB288382 and AB295051) were used as outgroup. Bootstrap support values for the NJ and ML trees were calculated in 500 replicates. The resulting consensus tree with the highest likelihood was obtained from a heuristic search by applying NJ and BioNJ algorithms to a matrix of pairwise distances estimated using the maximum composite likelihood approach. The rate variation model allowed for some sites to be evolutionarily invariable and the tree was drawn to scale, with branch lengths reflecting the number of substitutions per site. The analysis involved 46 nucleotide sequences (35 from GenBank database and 11 obtained from this study), for a total of 566 positions. NJ and ML analyses led to the same tree topology. The 11 sequences obtained in the present study were submitted to GenBank and can be accessed under the following accession numbers: LC438746 to LC438753 and KU160136 to KU160138.

## Results

### Description and Abundance of *M. polykrikoides* Planktonic Cells

**FIGURE 2:**
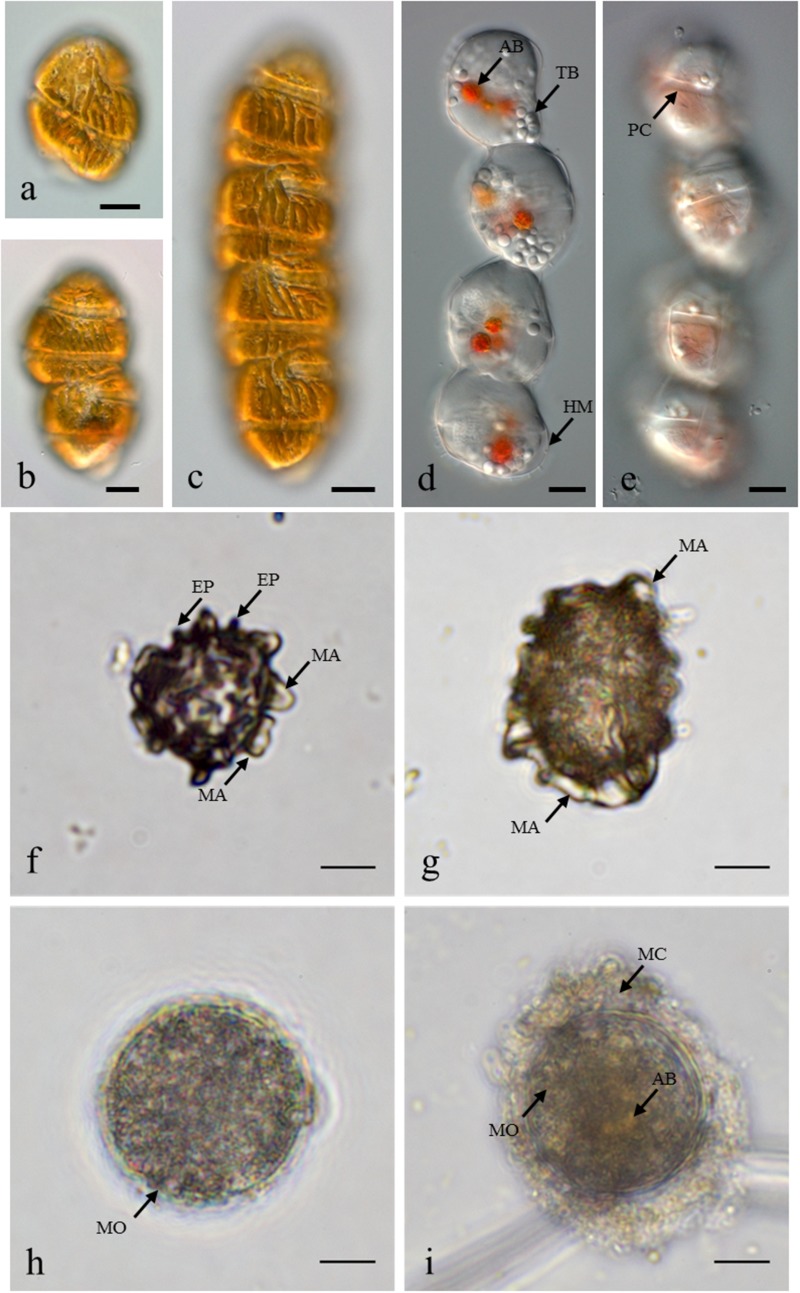
*Margalefidinium* planktonic cell and cyst morphotypes observed at Lampung Bay. **(a–c)** *M. polykrikoides* planktonic cells collected from D3 St1 in October 2014: **(a)** Single cell, **(b)** Two-cell chain, **(c)** four-cell chain; **(d,e)** *M. polykrikoides* hyaline cysts collected in August 2016. Scale bars = 10 μm. **(f–i)** *M. polykrikoides* cyst morphotypes isolated from surface sediment: “Matsuoka and Fukuyo-like” morphotype; apical view **(f)** and lateral view **(g)**; “Tang and Gobler-like” morphotype **(h)**; “Thoha-like” morphotype **(i)**. AB, accumulation body; TB, transparent body; HM, hyaline membrane; PC, paracingulum-like furrow; MA, machichorate; EP, evexate process; MO, microgranular ornementation; MC, mucilaginous coating. Scale bars = 10 μm.

In May 2014, the presence of *M. polykrikoides* vegetative cells in the water column was recorded at five stations: D1 St1, St3 and D2 St5, St7, St8. Cell concentrations varied from 2 to 26.2 cells L^-1^, with a maximum density at D2 St8 (Figure 1B and Supplementary Table S2). *M. polykrikoides* vegetative cells were also found in October 2014 at stations D1 St3, D2 St2, and D3 St1. Both single cells, two-cell and four-cell chains were observed (Figure 2a–c). However, the vast majority of motile cells were four-cell chains. In August 2016, temporary cysts, or hyaline cysts, were also found in the water column during a *M. polykrikoides* bloom at a density of 1.24 × 10^6^ cells L^-1^ at D3 St5 in Hurun Bay. Hyaline cysts were transparent, surrounded by a hyaline membrane, without chloroplasts and contained two to three red accumulation bodies and several spherical transparent bodies (Figure 2d). A paracingulum-like furrow structure was observed at the surface of hyaline cysts (Figure 2e). These hyaline cysts were associated with four-cell chains.

### Description, Distribution, and Abundance of *M. polykrikoides* Cyst Morphotypes

*Margalefidinium polykrikoides* cysts were detected in 17 of the 21 stations investigated in May 2014, and three main morphotypes were observed (Figure 2f–i). The first morphotype was quite similar to that described by Matsuoka (1985) and Matsuoka and Fukuyo (2000, 2003). This morphotype was a dark brown ovoid or ellipsoid (real shape or depending on the position in the Sedgwick rafter cell) proximochorate to chlorate cyst with an irregular shape (21–35 μm long and 15–27 μm wide). Its reticulated wall displayed a machichorate (membranous ornamentation) combined with thick evexate processes (Figure 2f,g). The second morphotype had perfect spherical and circular shape with a diameter of 25–41 μm. This greenish-brown color morphotype presented a thin cyst wall (with maybe two layers) with a smooth surface. In many cases, it exhibited a microgranular ornamentation. Archeopyle was not visible. This morphotype with a rough surface matched with the description of Tang and Gobler (2012; Figure 2h). A variant previously described by Thoha et al. (2015) was also observed. Its cyst wall was formed by a double layer with a microgranular membranous ornamentation and a mucilaginous coating around the cyst. The cyst contained a yellow-red accumulation body (Figure 2i). At least one *M. polykrikoides* cyst morphotype was recorded in all but four stations: D1 St1, St3, and St4, and D2 St5, which were among the deepest sampling points of the inner bay (Figure 1B and Table 2). Eleven of the 21 stations showed “Matsuoka and Fukuyo-like” cysts (MF-cysts), 14 stations showed “Tang and Gobler-like” cysts (TG-cysts), and its variant “Thoha-like” (T-like), and 11 stations showed at least two morphotypes (Table 2). The highest abundances of *M. polykrikoides*-like cysts were recorded off Bandar Lampung (D2 St2: 645.6 cysts g^-1^ DS, D2 St1: 172.7 cysts g^-1^ DS), and in Pandan and Hurun Bays (D3 St6: 174.8 cysts g^-1^ DS, D3 St7: 110.9 cysts g^-1^ DS) (Figure 1B and Supplementary Table S2).

**Table 2:** Relative abundance of the three morphotypes observed for *M. polykrikoides*-like cysts sampled at the 21 sampling stations in Lampung Bay.

Stations	*M. polykrikoides* cyst morphotypes (%)		
	“Matsuoka and Fukuyo-like”	“Tang and Gobler-like”	“Thoha-like”
D1 St1	0	0	0
D1 St2	50	0	50
D1 St3	0	0	0
D1 St4	0	0	0
D1 St5	30.8	0	69.2
D1 St7	100	0	0
D2 St1	0	4.7	95.3
D2 St2	3.4	0	96.6
D2 St3	0	0	100
D2 St4	3.7	0	96.3
D2 St5	0	0	0
D2 St6	75	0	25
D2 St7	80	20	0
D2 St8	0	0	100
D3 St1	0	100	0
D3 St2	71.4	14.3	14.3
D3 St3	100	0	0
D3 St4	20	0	80
D3 St5	0	25	75
D3 St6	40	0	60
D3 St7	0	0	100

### Molecular Identification of *M. polykrikoides* From Vegetative Cells and From Cyst Extracts

**FIGURE 3:**
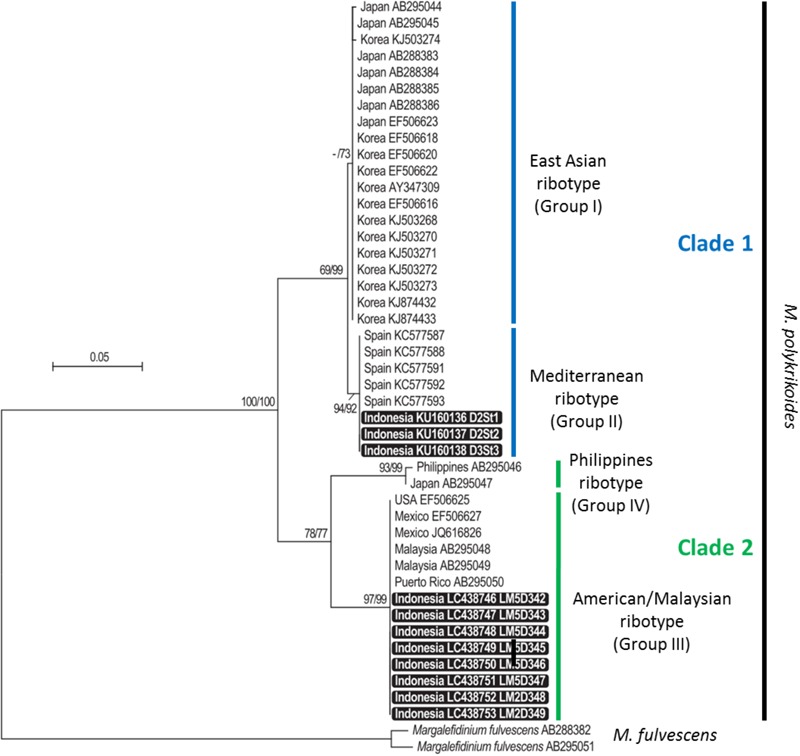
Maximum likelihood (ML) and neighbor-joining (NJ) tree based on partial LSU rDNA sequences of *Margalefidinium polykrikoides*. Bootstrap support values (indicated when ≥50) are shown at each node, for both NJ and ML topologies (NJ/ML). Sequences obtained in this study are highlighted in black boxes.

Partial LSU rDNA sequences (D1–D3 region, 1,497–1,523 bp) were obtained from the eight monoclonal cultures LM5D342, 343, 344, 345, 346, 347 (vegetative cells isolated at D3 St1) and LM2D348 and LM2D349 (vegetative cells isolated at D2 St2). Regarding cyst extracts, only three of them could be reliably amplified and sequenced (D1–D2 region, 566–581 bp), corresponding to stations D2 St1, D2 St2 and D3 St3. These amplicons were obtained with the primer combination D1R-Cpoly-R3. In order to compare them, the 11 sequences were aligned only on their common portion, representing a fragment of nearly 560 bp. The phylogenetic analysis, conducted with 35 already published sequences representing the four ribotypes identified thus far (East-Asian, Philippines, American-Malaysian, and Mediterranean clades), showed that the new sequences belonged to two different groups: those obtained from water samples clustered in the American-Malaysian clade, and sequences obtained from cyst extracts were closely related to the Mediterranean ribotype (Figure 3). The tree topology, supported by high bootstrap values, indicated a clear separation between the American-Malaysian, Philippines and East-Asian ribotypes, whereas the so-called Mediterranean ribotype tended to form a subgroup within the East-Asian clade.

## Discussion

Although *M. polykrikoides* blooms had been reported in the neighboring countries of Malaysia and Brunei in 2003, 2004, and 2005, and along the western coast of Palawan (Philippines) in 2005 (Azanza et al., 2008), *M. polykrikoides* had never been recorded in Indonesian waters prior to the first bloom that took place in Lampung Bay in October 2012. In the only survey performed in this area, no *M. polykrikoides* cyst was described in bottom sediment collected in Hurun Bay in 2003 (Mizushima et al., 2007). The present study is the first one that unequivocally reports the presence of the species *M. polykrikoides* in Indonesia, in the form of vegetative cells, hyaline cysts and resting cysts.

*M. polykrikoides* cysts were found in all studied sites of Lampung Bay, with the exception of D1 St1, D1 St3, D1 St4, and D2 St5, which were the deepest and sloping sampling points in the middle of the inner bay. Moreover, *M. polykrikoides* cyst concentrations, which were greater in the northern area of Lampung Bay, are among the highest densities ever reported along the East-Asian and Indian coasts (Wang et al., 2004; Orlova and Morozova, 2009; Shin et al., 2011; D’Silva et al., 2012). Many studies have pointed out that cysts play a decisive role in the ecology, population dynamics and spread of dinoflagellate species (Genovesi-Giunti et al., 2006; Penna et al., 2010; Bravo and Figueroa, 2014). However, the fact that significant densities of *M. polykrikoides* cysts are present in the sediments from which the blooms seem to originate does not constitute a direct evidence that recurrent blooms are due to these cysts. On the other hand, vegetative cells from Korean populations with an East-Asian ribotype have been shown to form temporary cysts surrounded by a hyaline membrane under certain environmental conditions (Kim et al., 2002). In the present study, hyaline cysts of *M. polykrikoides* were also found from the water column, in the form of four-cell chains, characteristics of the American-Malaysian ribotype. This transient form, capable of rapidly turning into vegetative cells by means of a luminous stimulus, seems to be a strategy for maintaining populations during blooms (Shin et al., 2017b).

Among the three main cyst morphotypes that were observed, the T-like one was predominant in sediments with the highest *M. polykrikoides* cyst densities (Table 2). Such a mucilaginous layer around thornless morphotypes may allow the cysts to aggregate with other particles and thus become denser (Bravo and Figueroa, 2014). As was shown for other taxonomic groups, cyst morphological changes can be considered as indicators of environmental variations (e.g., eutrophication, acidification, salinity or temperature change) (Ellegaard, 2000; Mertens et al., 2009; Rochon et al., 2009; Persson et al., 2013; Shin et al., 2013). D2 St1, 2, 3, D2 St8, and D3 St6–7, which are the most affected by regular blooms, correspond to shallow and highly anthropized stations, very close to the shore and river mouths. At these stations, local hypoxic conditions in the bottom sediment due to terrestrial organic inputs and freshening are quite common (Damar, 2003; Santoso and Hayami, 2005). Thus, the hypothesis that morphotype varies according to geochemical conditions in the water column during cyst formation and its residence time in the surface sediment is also plausible here. Ornamented cysts could therefore be more recent or generated under conditions that do not inhibit the formation of the reticulated wall with membranous ornamentation. No ornamented cyst morphotype with thick spines and reticulate ornaments as described by Li et al. (2015), nor the round morphotype with a concave and flat side (Kim et al., 2007), were observed in the present samples.

Obtaining vegetative cells from cyst germination is an essential step in the taxonomic identification of species with a dormant stage. Unfortunately, despite our efforts, germination experiments on single cysts and natural sediments from “hotspot” *M. polykrikoides* bloom areas and high *M. polykrikoides* cyst concentration areas were unsuccessful. In most studies describing *M. polykrikoides* resting cyst morphotypes from natural sediments, species identification could not be achieved because cyst germination was not realized and/or successful. As far as we know, only two studies have been able to obtain cyst germination from environmental samples and to formally identify the species: Mohamed and Al-Shehri (2011) and Li et al. (2015). The most widely reported cyst morphotype is the MF one described by Matsuoka (1985) and Matsuoka and Fukuyo (2000) (Wang et al., 2004; Orlova and Morozova, 2009; Mohamed and Al-Shehri, 2011; D’Silva et al., 2012). This ornamented morphotype was considered as the resting stage corresponding to *M. polykrikoides* strains belonging to the East-Asian ribotype. However, different morphotypes were isolated from Korean sediment (Kim et al., 2007; Li et al., 2015). Kim et al. (2007) described a round morphotype with a concave and flat side from culture and natural sediment samples, while Li et al. (2015) described a brown cyst with spines, containing granular contents and a red accumulation, which gave vegetative cells similar to those found in the water column after germination. *M. polykrikoides* belonging to the East-Asian ribotype may therefore naturally display distinct morphotypes. Furthermore, Tang and Gobler (2012) generated spherical resting cysts from *in vitro* sexual reproduction of American-compatible strains and Hattenrath-Lehmann et al. (2016) described a similar morphotype in a FISH assay from environmental sediments sampled in the northeastern United States. Therefore, *M. polykrikoides* ribotypes may have several resting cyst morphotypes that may depend on genetic factors inherent to a given ribotype/clade and/or on environmental factors (e.g., pH, dissolved oxygen, salinity, temperature) occurring during the planktonic stage and/or during the resting period. So far, only few studies could establish a clear relationship between morphotype and ribotype, and we cannot rule out that the different cyst morphotypes observed could also belong to several species which would then be regarded as cryptic. This could be supported by the facts that *Margalefidinium fulvescens* has been formally described in Hurun bay (Iwataki et al., 2007) and that these two species are phylogenetically close (Gómez et al., 2017). The taxonomic clarification of this genus will require more investigations, as was the case within *Alexandrium* genus for *Alexandrium catenella, Alexandrium tamarense, Alexandrium fundyense*, and *Alexandrium pacificum* (John et al., 2014; Fraga et al., 2015; Prud’homme, 2017; Shin et al., 2017a).

Molecular detection of *M. polykrikoides* performed on genomic DNA isolated from motile cells and cyst extracts revealed the presence of two ribotypes in Lampung Bay. The American-Malaysian ribotype was detected from motile cells isolated in the water column in October 2014. The Mediterranean ribotype was found in three cyst extracts from sediment harvested from locations where several *M. polykrikoides* cyst morphotypes coexisted, at stations D2 St1 and St2 (where the T-like morphotype was dominant) and at station D3 St3 (where the MF-like morphotype was dominant) (Table 2). However, our results do not allow to assign a cyst morphotype to a ribotype because of the unsuccessful germination. The fact that we did not detect any Mediterranean ribotype from vegetative cells, which is consistent with the over-dominance of four-cell chains in the water column, could indicate that this ribotype is part of the “sediment seed” that does not germinate under the environmental conditions of Lampung bay during the sampling period. On the other hand, in spite of our efforts, only three sequences from sediment cyst extracts could be obtained, all belonging to the Mediterranean ribotype. Considering the difficulty to amplify single sequences from sediment cyst extracts, mainly because of the existence of multiple PCR inhibitors (Park et al., 2014), these sequences are not likely to reflect the ribotype diversity that may exist in the sediments of Lampung Bay, and do not preclude the presence of the American-Malaysian ribotype. Though it is not devoid of experimental biases (e.g., false positives or false negatives), DNA metabarcoding would be the most reliable way to sort out this question (Smith et al., 2017). We intend to apply this method on both sediment and water samples in the future studies on Lampung Bay.

The phylogenetic analysis suggested that the so-called Mediterranean ribotype was rather a subgroup of the East-Asian cluster than a specific clade. As already proposed by Reñé et al. (2013), it seems more accurate to name ribotypes by numbered groups and clades rather than by geographical origin, and use the following nomenclature: clade 1 for East-Asian (Group I) and Mediterranean (Group II) ribotypes, clade 2 for American-Malaysian (Group III) and Philippines (Group IV) ribotypes. Only sampling efforts and ribotyping of many individuals (vegetative cells, hyaline, and resting cysts) and of environmental samples will make it possible to understand the structuration of the ribotypes within this species. This approach was used to describe the diversity within the *A. tamarense* complex (Lilly et al., 2007; Genovesi et al., 2011). Our findings are comparable to those reported by Park et al. (2014, 2016, 2018), who detected two ribotypes in the water column [the Philippines (clade IV) and East-Asian (clade I) ribotypes] and to those of Reñé et al. (2013) who found the Philippines ribotype accompanied by the novel Mediterranean ribotype. Nagai et al. (2009) described in detail the genetic diversity of this species on Japanese coasts using microsatellite markers. They highlighted three major populations and hypothesized contamination by pearl oyster transport during the 1980–1990s. A Japanese pearl oyster (*Pinctada maxima*) farm was established in 1986 at station D3 St4 over an area of 100 ha. Thus, in addition to ballast water discharge, shellfish transfers can be a source of contamination, and further analysis is needed to test the hypothesis of an expansion through shellfish transfers in Lampung Bay.

## Author Contributions

EM, HT, M, TS, AR, and MI conceived and designed the study. EM, HT, M, TS, AR, MI, and KT performed the field sampling. EM, MBI, AR, HT, TS, M, MI, and KT performed the taxonomical identifications. EM, MBI, MI, KT, and J-CA performed the molecular work. EM, M, MBI, OS, MI, KT, and J-CA analyzed the data, wrote the manuscript, prepared the figures and/or tables. HT and AR revised the draft of the manuscript.

## Conflict of Interest Statement

The authors declare that the research was conducted in the absence of any commercial or financial relationships that could be construed as a potential conflict of interest.

## Footnotes

1 https://www.marum.de/Karin-Zonneveld/dinocystkey.html
