# Supplementary material for: Resting Cyst Distribution and Molecular Identification of the Harmful Dinoflagellate Margalefidinium polykrikoides (Gymnodiniales, Dinophyceae) in Lampung Bay, Sumatra, Indonesia
Source: Front Microbiol. 2019 Feb 21;10:306. doi: 10.3389/fmicb.2019.00306 (PMC6393338; doi:10.3389/fmicb.2019.00306)
Supplement: Supplementary file 1 [file Table_1.DOCX]

Table S1 GPS coordinates, depth and abiotic parameters (temperature, pH, dissolved oxygen concentrations, salinity and sediment water content) of the 21 stations sampled in May 2014 at Lampung Bay (Sumatra, Indonesia).

| Stations | GPS coordinates | | Depth | Temperature | pH | Dissolved oxygen | Salinity | Water content |
| --- | --- | --- | --- | --- | --- | --- | --- | --- |
|  | South | East | (m) | (°C) |  | (mg L-1) |  | (%) |
| D1 St1 | -05.55649 | 105.30917 | 27 | 30.2 | 8 | - | 32 | 8.38 |
| D1 St2 | -05.55984 | 105.35947 | 23 | 31 | 8 | 7.45 | 32 | 8.61 |
| D1 St3 | -05.57970 | 105.31030 | 27 | 32.4 | 8.4 | 7.48 | 32 | 10.62 |
| D1 St4 | -05.61425 | 105.31351 | 20 | 32 | 8.4 | 8.07 | 33 | 8.96 |
| D1 St5 | -05.60369 | 105.17780 | 11 | 31 | 8.2 | 7.97 | 31 | 8.00 |
| D1 St7 | -05.59577 | 105.23958 | 20 | 32 | 8.4 | 8.09 | 32 | 9.53 |
| D2 St1 | -05.45220 | 105.28925 | 16 | 30.5 | 7.9 | 8.45 | 31 | 7.33 |
| D2 St2 | -05.46368 | 105.26250 | 3 | 31.4 | 7.8 | 7.66 | 26 | 7.84 |
| D2 St3 | -05.47179 | 105.31441 | 13 | 30.6 | 7.9 | 7.01 | 32 | 7.69 |
| D2 St4 | -05.47544 | 105.28833 | 24 | 30.1 | 8 | 8.39 | 32 | 7.60 |
| D2 St5 | -05.49941 | 105.29119 | 26 | 30.6 | 8.1 | 7.99 | 32 | 8.47 |
| D2 St6 | -05.51537 | 105.33084 | 25.1 | 30.5 | 8 | 8.29 | 33 | 7.38 |
| D2 St7 | -05.53187 | 105.30119 | 27.6 | 29.6 | 8.3 | 7.85 | 33 | 9.08 |
| D2 St8 | -05.48645 | 105.25816 | 9 | 31.3 | 8 | 8.19 | 31 | 9.97 |
| D3 St1 | -05.56728 | 105.24458 | 7.5 | 30.5 | 7.9 | 7.85 | 31 | 7.76 |
| D3 St2 | -05.56261 | 105.26290 | 27 | 30.2 | 8.1 | 7.96 | 32 | 8.40 |
| D3 St3 | -05.53870 | 105.25866 | 21 | 30.6 | 8.1 | 7.25 | 32 | 7.40 |
| D3 St4 | -05.53187 | 105.27290 | 26 | 30.5 | 8.1 | 8.03 | 33 | 8.23 |
| D3 St5 | -05.52331 | 105.25093 | 12 | 30.8 | 8 | 7.49 | 31 | 8.27 |
| D3 St6 | -05.54335 | 105.25053 | 14 | 30.6 | 8 | 7.29 | 32 | 9.42 |
| D3 St7 | -05.51705 | 105.24992 | 3.5 | 29.8 | 7.9 | 6.48 | 31 | 8.59 |

Table S2: *M. polykrikoides* vegetative cell and *M. polykrikoides*-like cyst abundances at the 21 stations sampled in May 2014 at Lampung Bay (Sumatra, Indonesia).

| Stations | *M. polykrikoides* cells | *M. polykrikoides* cysts |
| --- | --- | --- |
|  | (cell L^-1^) | (cysts g^-1^ DS) |
| D1 St1 | 2.0 | 0.0 |
| D1 St2 | 0.0 | 7.3 |
| D1 St3 | 2.0 | 0.0 |
| D1 St4 | 0.0 | 0.0 |
| D1 St5 | 0.0 | 47.1 |
| D1 St7 | 0.0 | 29.5 |
| D2 St1 | 0.0 | 172.7 |
| D2 St2 | 0.0 | 645.6 |
| D2 St3 | 0.0 | 20.6 |
| D2 St4 | 0.0 | 92.5 |
| D2 St5 | 6.1 | 0.0 |
| D2 St6 | 0.0 | 13.7 |
| D2 St7 | 4.1 | 17.4 |
| D2 St8 | 26.2 | 24.6 |
| D3 St1 | 0.0 | 28.9 |
| D3 St2 | 0.0 | 45.8 |
| D3 St3 | 0.0 | 54.7 |
| D3 St4 | 0.0 | 23.0 |
| D3 St5 | 0.0 | 55.2 |
| D3 St6 | 0.0 | 174.8 |
| D3 St7 | 0.0 | 110.9 |
